# Non-Invasive Preoperative Imaging Differential Diagnosis of Intracranial Hemangiopericytoma and Angiomatous Meningioma: A Novel Developed and Validated Multiparametric MRI-Based Clini-Radiomic Model

**DOI:** 10.3389/fonc.2021.792521

**Published:** 2022-01-04

**Authors:** Yanghua Fan, Panpan Liu, Yiping Li, Feng Liu, Yu He, Liang Wang, Junting Zhang, Zhen Wu

**Affiliations:** ^1^ Department of Neurosurgery, Beijing Tiantan Hospital, Capital Medical University, Beijing, China; ^2^ Department of Neurosurgery, Beijing Neurosurgical Institute, Beijing, China; ^3^ Department of Neurosurgery, Weihai Municipal Hospital, Cheeloo College of Medicine, Shandong University, Weihai, China; ^4^ Department of Gastroenterology, Weihai Municipal Hospital, Cheeloo College of Medicine, Shandong University, Weihai, China; ^5^ Department of Neurosurgery, Jiangxi Provincial Children’s Hospital, The Affiliated Children’s Hospital of Nanchang University, Nanchang, China; ^6^ Department of Craniomaxillofacial Surgery, Plastic Surgery Hospital, Chinese Academy of Medical Sciences & Peking Union Medical College, Beijing, China

**Keywords:** intracranial hemangiopericytoma, angiomatous meningioma, radiomics, algorithm, diagnosis

## Abstract

**Background:**

Accurate preoperative differentiation of intracranial hemangiopericytoma and angiomatous meningioma can greatly assist operation plan making and prognosis prediction. In this study, a clini-radiomic model combining radiomic and clinical features was used to distinguish intracranial hemangiopericytoma and hemangioma meningioma preoperatively.

**Methods:**

A total of 147 patients with intracranial hemangiopericytoma and 73 patients with angiomatous meningioma from the Tiantan Hospital were retrospectively reviewed and randomly assigned to training and validation sets. Radiomic features were extracted from MR images, the elastic net and recursive feature elimination algorithms were applied to select radiomic features for constructing a fusion radiomic model. Subsequently, multivariable logistic regression analysis was used to construct a clinical model, then a clini-radiomic model incorporating the fusion radiomic model and clinical features was constructed for individual predictions. The calibration, discriminating capacity, and clinical usefulness were also evaluated.

**Results:**

Six significant radiomic features were selected to construct a fusion radiomic model that achieved an area under the curve (AUC) value of 0.900 and 0.900 in the training and validation sets, respectively. A clini-radiomic model that incorporated the radiomic model and clinical features was constructed and showed good discrimination and calibration, with an AUC of 0.920 in the training set and 0.910 in the validation set. The analysis of the decision curve showed that the fusion radiomic model and clini-radiomic model were clinically useful.

**Conclusions:**

Our clini-radiomic model showed great performance and high sensitivity in the differential diagnosis of intracranial hemangiopericytoma and angiomatous meningioma, and could contribute to non-invasive development of individualized diagnosis and treatment for these patients.

## Introduction

Intracranial hemangiopericytoma is a rare mesenchymal tumor with strong aggressiveness and high degree of vascularization (1). Many features of intracranial hemangiopericytoma are similar to meningioma; intracranial hemangiopericytoma and meningioma both originate from the meninges and have similar imaging features. In particular, angiomatous meningioma with invasive image but benign behavior is difficult to distinguish from intracranial hemangiopericytoma before operation. However, angiomatous meningioma and intracranial hemangiopericytoma have different histological characteristics and biological behaviors (2). Angiomatous meningioma is a rare benign variant tumor classified as WHO grade 1 meningioma. Compared with angiomatous meningiomas, intracranial hemangiopericytoma is classified as a malignant tumor (WHO grades II–III) with the tendency of recurrence and metastasis (3). Intracranial hemangiopericytoma is more aggressive, highly vascularized and prone to intraoperative hemorrhage, has a higher postoperative recurrence rate and a worse prognosis (1).

More preoperative preparation is needed to ensure the maximum surgical resection and safety for intracranial hemangiopericytoma, such as more detailed surgical strategy, preoperative tumor feeding artery embolization, the use of intraoperative navigation equipment, and more adequate spare blood (4, 5). Therefore, their preoperative preparation and treatment principles are largely different thus the preoperative differential diagnosis is crucial. The high overlap of the radiological characteristics of intracranial hemangiopericytoma and angiomatous meningioma poses a great challenge to the preoperative imaging identification (2). Although intracranial hemangiopericytoma is usually male and the age of onset is relatively early, it is difficult to diagnose based on this (6). Previous studies have shown that radiomic features may help distinguish intracranial hemangiopericytoma from meningioma (7), but more attention should be paid to the identification of angiomatous meningioma and intracranial hemangiopericytoma, which are more likely to be confused in the clinic.

Radiomics emerges as a potent approach for the non-invasive high-throughput mining of tumor characteristics (8, 9). Neuro-oncologic radiomic studies can potentially mine the hidden data that cannot be obtained through single-parameter and conventional imaging approach; meanwhile, they can also enhance the accuracy and effectiveness in the differential diagnosis for intracranial tumor (10–12). Due to a relatively low incidence, previous studies (13) on the identification of angiomatous meningioma and intracranial hemangiopericytoma have many shortcomings such as the small number of patients included and no verification set. Consequently, based on a relatively large number of patients, the purpose of this study is to establish a clini-radiomic combined model that combines radiomic and clinical features to distinguish intracranial hemangiopericytoma and angiomatous meningioma before surgery, and to assist in preoperative planning for the management and treatment of the two types of tumors.

## Materials and Methods

### Patients

A total of 220 patients (Angiomatous meningioma: n = 73; Hemangiopericytoma: n = 147) were included from the Beijing Tiantan Hospital, Capital Medical University. The inclusion criteria of enrolled patients in this study were as follows: 1) intracranial hemangiopericytoma or atypical meningioma patients who underwent initial tumor resection surgery from 2010 to 2019 at the Beijing Tiantan Hospital; 2) available postoperative pathological diagnosis results information; 3) patient underwent preoperative head T2-weighted imaging (T2WI) and contrast-enhanced T1-weighted imaging (CE-T1WI) MRI examination; and 4) complete clinical information at initial diagnosis. All included patients were randomized to the training set (used for model building, n = 147) and validation set (used for model validation, n = 73) at a ratio of 2:1.

### Clinical Characteristics

Seven preoperative clinical characteristics from all included patients were collected: age, gender, location 1 (supratentorial or infratentorial), location 2 (skull base or non-skull base), location 3 (paravenous sinus or non-paravenous sinus), dural tail (negative or positive), and peritumoral serious edema (negative or positive). Moreover, the patient’s postoperative pathological results determine whether it is angiomatous meningioma or hemangiopericytoma that needs to be collected. According to the WHO pathology guidelines (3), angiomatous meningioma is defined as meningioma with >50% vascular components (14).

### Regions of Interest (ROI) Delineating and Radiomic Feature Extraction

The flowchart and scheme of this study are similarly described in detail in our previous researches (15, 16). All patients underwent preoperative brain T2WI and CE-T1WI MR imaging. CE-T1WI was carried out the T1WI sequence parameters after rapid injection of a gadolinium-DTPA contrast agent. A neuroradiologist with 8 years of experience used ITK-SNAP software to map the three-dimensional ROIs of the tumor on T2WI and CE-T1WI MR imaging. Then, another neurosurgeon with 13 years of experience reviewed, modified, and confirmed the above segmentation results. Any disagreements between the two neuroradiologists are resolved through mutual consultation.

Then, the PyRadiomics algorithm (Version 2.1.2; https://github.com/Radiomics/pyradiomics) was used to extract quantitative four types of 1,562 radiomic features from the above segmented ROI, all features are standardized to a value of 0 to 1 (12, 17). The four types of features were described as follows (15, 18): 1) shape and size features (n = 14) were independent of the gray-scale intensity distribution of the tumor; 2) the first-order statistics (n = 180) described the distribution of voxel intensity in the image through basic metrics; 3) Texture features (n = 680) are calculated from the gray-level co-occurrence matrix (GLCM) and gray-level run-length matrix (GLRLM), respectively, to describe the pattern or the spatial distribution of voxel intensity; and 4) Wavelet features (n = 688) transform effectively decouples textural information by decomposing the original image at low and high frequencies in a manner similar to Fourier analysis.

### Radiomic Features Selection and Fusion Radiomic Model Construction

After radiomic feature extraction, a selection process is adopted to reduce overfitting (19). First, the Mann–Whitney U test was used to determine the significantly different radiomic features of patients with intracranial hemangiopericytoma and atypical meningioma. Then, the elastic net algorithm (20), a method that combines the minimum absolute shrinkage and selection operator (LASSO) and ridge regression, was used to select the most informative features. LASSO is a commonly used high-dimensional data analysis method, which can improve the prediction accuracy and interpretation ability (21). Finally, recursive feature elimination (RFE) is used to determine the final radiomic features through a five-time cross-validation algorithm.

Through the support vector machine (SVM) method of training set, a T1 radiomic model, a T2 radiomic model, and a fusion radiomic model were constructed from the meaningful features selected from the separate CE-T1WI radiomic feature, separate T2WI radiomic features, and mixed CE-T1WI and T2WI radiomic features, respectively.

### Construction and Validation of Clinical and Clini-Radiomic Model

Multivariable logistic regression analysis was applied to construct a clinical model based on all included clinical features. Then, to establish a more accurate and comprehensive model for discriminating the hemangiopericytoma and angiomatous meningioma, a clini-radiomic model was constructed by combining the above clinical model with the fusion radiomic model. The structure and parameters of the clini-radiomic model was presented as a nomogram.

### Calibration Curve Analysis and Decision Curve Analysis

Calibration curves and the Hosmer–Lemeshow test were used to assess the similarity between the observed pathological results and predicted diagnosis results of fusion radiomic model and clini-radiomic model (22). Decision curve analysis (DCA) was performed to evaluate the clinical application of the fusion radiomic model and clini-radiomic model by quantifying the net benefits at different threshold probabilities (23).

### Statistical Analysis

A two-sided *P*-value of <0.05 was deemed to be statistically significant. Categorical variables were presented as the number (percentage). Continuous variables consistent with a normal distribution were presented as mean ± standard deviation, otherwise the median and quartile are used. Chi-Square test was used to compare the differences in categorical variables. The independent sample t-test was used to compare the differences in continuous variables that conform to the normal distribution, otherwise the nonparametric test was used to compare the differences in continuous variables with non-normal distribution.

The statistical software R (version 3.4.1, R Foundation for Statistical Computing, Vienna, Austria) was used to perform the statistical analysis. The violin plot algorithm was used to show the differences in the signature distribution of fusion radiomic model between intracranial hemangiopericytoma and atypical meningiomas in the training set and validation set. The receiver operating characteristic (ROC) curve was used to show the predicted value of the above constructed models (24). The calibration plot was analyzed with the ‘hdnom’ packages. The decision curve analysis is performed by the “dca.R: function written by us in the software R. The DeLong’s test was used to compare the prediction performance differences of the constructed models.

## Results

### Clinical Characteristics

According to the inclusion and exclusion criteria, 220 patients with intracranial hemangiopericytoma or angiomatous meningioma were identified and included in this study. The mean age at diagnosis was 49.0 (37.0–55.0) years, with a female-to-male ratio of 1.157:1 (118/102). Of the 220 patients, 80 (36.4%) patients had peritumoral edema, and 29 (13.2%) patients had dural tail in CET1 images. A total of 147 (66.8%) patients were pathologically diagnosed as intracranial hemangiopericytoma and 73 (33.2%) patients were diagnosed as angiomatous meningioma. All included clinical characteristics are summarized in **Table 1**.

**Table 1:** Patients’ characteristics of training and validation sets.

Characteristics	All sets (n = 220)	Training set (n = 147)	Validation set (n = 73)	P-value
**Age (year)**	49.0 (37.0–55.0)	49.0 (35.0–56.0)	49.0 (40.0–54.0)	0.900
**Gender**				
Female	118 (53.6%)	79 (53.7%)	39 (51.5%)	0.965
Male	102 (46.4%)	68 (46.3%)	34 (48.5%)	
**Location 1**				
Supratentorial	181 (82.3%)	117 (79.6%)	64 (87.7%)	0.140
Infratentorial	39 (17.7%)	30 (20.4%)	9 (12.3%)	
**Location 2**				
Non skull base	152 (69.1%)	103 (70.1%)	49 (67.1%)	0.656
Skull base	68 (30.9%)	44 (29.9%)	24 (32.9%)	
**Location 3**				
Non paravenous sinus	111 (50.5%)	70 (47.6%)	41 (56.2%)	0.233
Paravenous sinus	109 (49.5%)	77 (52.4%)	32 (43.8%)	
**Dural tail**				
Negative	191 (86.8%)	131 (89.1%)	60 (82.2%)	0.153
Positive	29 (13.2%)	16 (10.9%)	13 (17.8%)	
**Peritumoral edema**				
Negative	140 (63.6%)	96 (65.3%)	44 (60.3%)	0.465
Positive	80 (36.4%)	51 (34.7%)	29 (39.7%)	
**Diagnosis**				
Angiomatous meningioma	73 (33.2%)	49 (33.3%)	24 (32.9%)	0.946
Hemangiopericytoma	147 (66.8%)	98 (66.7%)	49 (67.1%)	

All patients were randomly divided into a training set (n = 147) and a validation set (n = 73). There was no significant interclass difference in terms of age (P = 0.900), gender (P = 0.965), locations 1, 2, and 3 (P = 0.140, 0.656, and 0.233), dural tail (P = 0.153), peritumoral edema (P = 0.465), and diagnosis (P = 0.946) between the training set and the validation set (**Table 1**). The results justify the use of the two datasets for training and testing.

### Univariate Analysis of Clinical Characteristics and Postoperative Pathological Diagnosis

As shown in **Table 2**, age, location 1, location 2, location 3, dural tail, and peritumoral edema showed significant relationships with postoperative pathological diagnosis (all P <0.05). The results demonstrated that elder patients who had infratentorial, non-skull base, paravenous sinus, dural tail or peritumoral serious edema tumor were more likely to have angiomatous meningioma. Conversely, we found no significant differences in gender (P = 0.965) between the intracranial hemangiopericytoma and angiomatous meningioma patients.

**Table 2:** Clinical characteristics of patients with intracranial hemangiopericytoma and angiomatous meningioma.

Characteristics	All patients (n = 220)	Angiomatous meningioma (n = 73)	Hemangiopericytoma (n = 147)	P-value
**Age (year)**	49.0 (37.0–55.0)	53.0 (44.5–59.0)	46.0 (35.0–53.0)	0.000
**Gender**				
Female	118 (53.6%)	39 (53.4%)	79 (53.7%)	0.965
Male	102 (46.4%)	34 (46.6%)	68 (46.3%)	
**Location 1**				
Supratentorial	181 (82.3%)	69 (94.5%)	112 (76.2%)	0.001
Infratentorial	39 (17.7%)	4 (5.5%)	35 (23.8%)	
**Location 2**				
Non skull base	152 (69.1%)	57 (78.1%)	95 (64.6%)	0.042
Skull base	68 (30.9%)	16 (21.9%)	52 (35.4%)	
**Location 3**				
Non paravenous sinus	111 (50.5%)	29 (39.7%)	82 (55.8%)	0.025
Paravenous sinus	109 (49.5%)	44 (60.3%)	65 (44.2%)	
**Dural tail**				
Negative	191 (86.8%)	51 (69.9%)	140 (95.2%)	0.000
Positive	29 (13.2%)	22 (30.1%)	7 (4.8%)	
**Peritumoral edema**				
Negative	140 (63.6%)	27 (37.0%)	113 (76.9%)	0.000
Positive	80 (36.4%)	46 (63.0%)	34 (46.3%)	

As shown in **Table 3**, univariate analysis was used to determine the independent clinical risk features for postoperative pathological diagnosis in the training and the validation sets, respectively. Similar to the previous results, in the training set, we found a significant association between pathological diagnosis and age (P = 0.007), location 1 (P = 0.001), location 2 (P = 0.030), location 3 (P = 0.010), dural tail (P = 0.009), and peritumoral edema (P <0.0001). In the validation set, age (P = 0.003), location 1 (P = 0.467), dural tail (P <0.0001), and peritumoral edema (P = 0.001) tended to be associated with pathological diagnosis.

**Table 3:** Univariate analysis of clinical characteristics of intracranial hemangiopericytoma and angiomatous meningioma patients in the training set and validation set.

Characteristics	Training set (n = 147)		P-value	Validation set (n = 73)		P-value
	Angiomatous meningioma (n = 49)	Hemangiopericytoma (n = 98)		Angiomatous meningioma (n = 24)	Hemangiopericytoma (n = 49)	
**Age (year)**	54.0 (43.0–58.5)	46.5 (32.75–54.25)	0.007	51.0 (47.5–60.75)	46.0(37.0-51.0)	0.003
**Gender**						
Female	25 (51.0%)	54 (55.1%)	0.640	14 (58.3%)	25 (51.0%)	0.556
Male	24 (49.0%)	44 (44.9%)		10 (41.7%)	24 (49.0%)	
**Location 1**						
Supratentorial	47 (95.9%)	70 (71.4%)	0.001	22 (91.7%)	42 (85.7%)	0.467
Infratentorial	2 (4.1%)	28 (28.6%)		2 (8.3%)	7 (14.3%)	
**Location 2**						
Non skull base	40 (81.6%)	63 (64.3%)	0.030	17 (70.8%)	32 (65.3%)	0.637
Skull base	9 (18.4%)	35 (35.7%)		7 (29.2%)	17 (34.7%)	
**Location 3**						
Non paravenous sinus	16 (32.7%)	54 (55.1%)	0.010	13 (54.2%)	28 (57.1%)	0.810
Paravenous sinus	33 (67.3%)	44 (44.9%)		11 (45.8%)	21 (42.9%)	
**Dural tail**						
Negative	39 (79.6%)	92 (93.9%)	0.009	12 (50.0%)	48 (98.0%)	0.000
Positive	10 (20.4%)	6 (6.1%)		12 (50.0%)	1 (2.0%)	
**Peritumoral edema**						
Negative	19 (38.8%)	77 (78.6%)	0.000	8 (33.3%)	36 (73.5%)	0.001
Positive	30 (61.2%)	21 (21.4%)		16 (66.7%)	13 (26.5%)	

### Radiomic Feature Selection and Radiomic Model Construction

**Figure 1:**
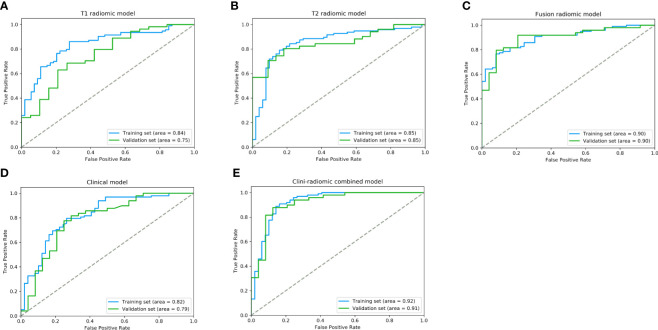
The performance of ROC curves for the predictive models the training and validation sets. **(A)** T1 radiomic model; **(B)** T2 radiomic model; **(C)** fusion radiomic model; **(D)** Clinical model; and **(E)** Clini-radiomic combined model.

Based on the extracted 1,562 CE-T1WI radiomic features, 262 radiomic features were selected by Mann–Whitney U test. Then, we use ‘elastic net’ algorithm to determine 21 informative features. Through the screening by RFE algorithm with 5-fold cross validation, 2 CE-T1WI radiomic features were selected as the final features for subsequent use. The selected two CE-T1WI radiomic features were entered into an SVM to build a T1 radiomic model, which showed discrimination in predicting the postoperative pathological diagnosis with AUC values of 0.840 (95% confidence interval [CI], 0.814–0.863) and 0.750 (95% CI, 0.718–0.788) in the training and validation sets, respectively (**Figure 1A**). Similar to the previous, 9 T2WI radiomic features were selected and then entered into an SVM to build a T2 radiomic model, with AUC value of 0.850 (95% CI, 0.829–0.879) in the training set and 0.850 (95% CI, 0.828-0.873) in the validation set (**Figure 1B**). The DeLong’s test showed that there was no significant difference between the T1 and T2 radiomic models (P = 0.275).

**Figure 2:**
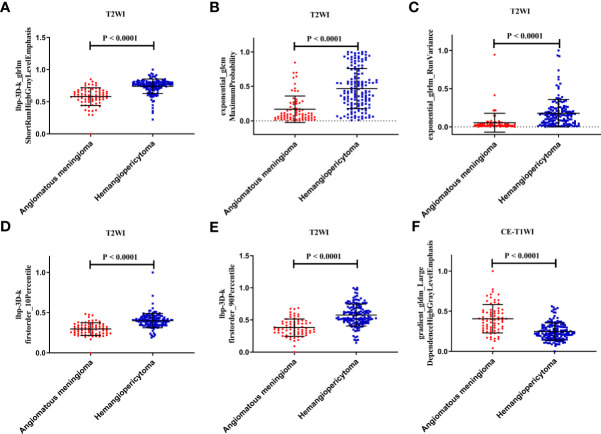
The six selected radiomic features had significant differences between patients with intracranial hemangiopericytoma and angiomatous meningiomas. **(A)** lbp-3D-k_glrlmShortRunHighGrayLevelEmphasis; **(B)** exponential_glcm MaximumProbability; **(C)** exponential_glrlm_RunVariance; **(D)** lbp-3D-k firstorder_10Percentile; **(E)** lbp-3D-k firstorder_90Percentile; and **(F)** gradient_gldm_Large DependenceHighGrayLevelEmphasis.

**Figure 3:**
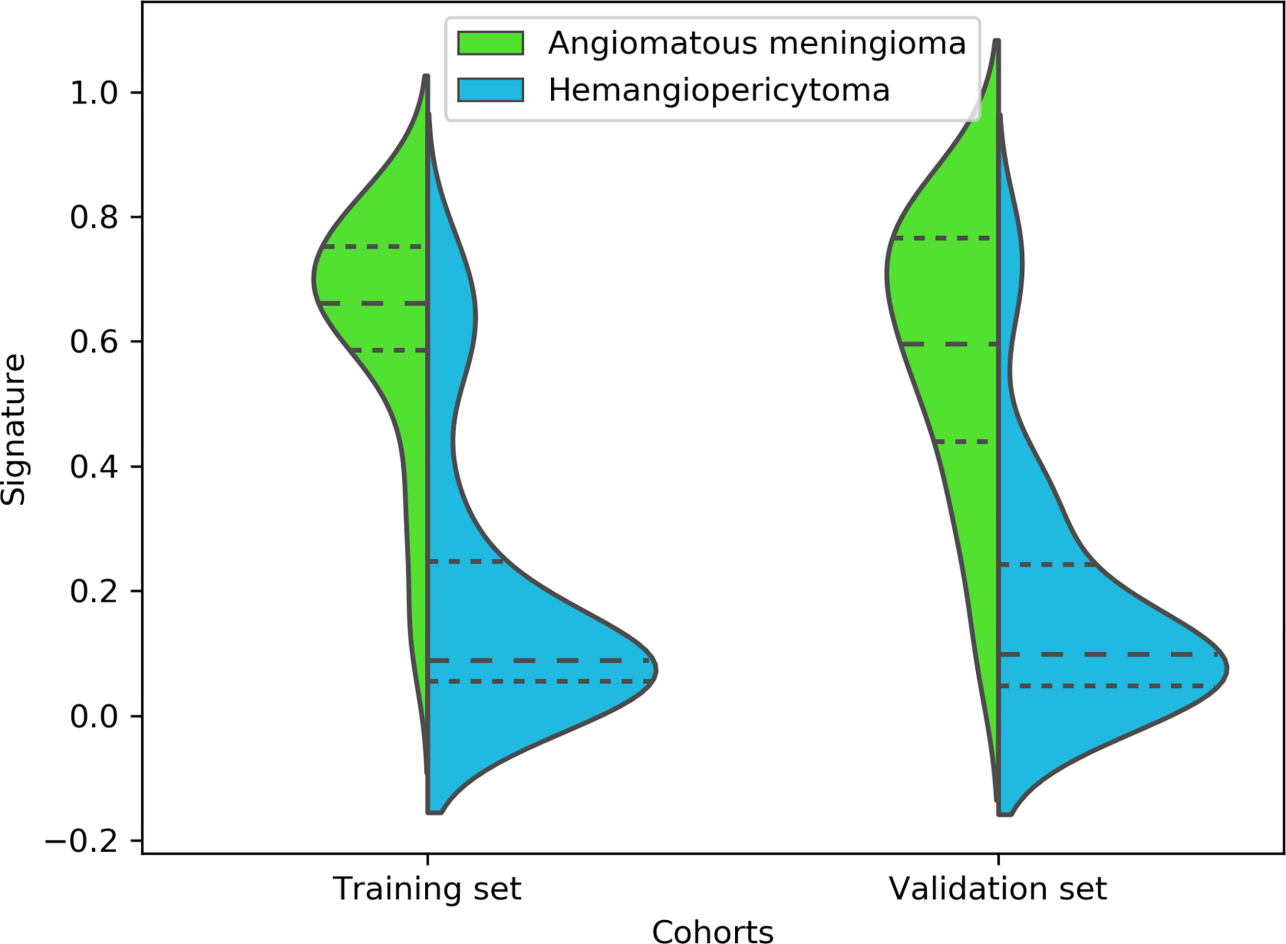
A violin plot showing the signature distribution of the fusion radiomic model between intracranial hemangiopericytoma and angiomatous meningioma patients.

Finally, among the 3,124 mixed CE-T1WI and T2WI radiomic features, 399 radiomic features were selected by Mann–Whitney U test. ‘Elastic net’ algorithm was used to determine 37 informative features. Finally, through the screening by RFE algorithm with 5-fold cross validation, 6 radiomic features (2 first order feature and 4 texture features) that gave the best performance were selected as the final features for subsequent use. Only 1 feature was selected from the CE-T1WI images, and 5 features from the T2WI images. All 6 selected radiomic features had significant differences between patients with intracranial hemangiopericytoma and angiomatous meningioma (All P <0.0001, **Table 4** and **Figure 2**). All 6 selected features were used to build a fusion radiomic model. The violin plot showed significant differences in the signature distribution of fusion radiomic model between intracranial hemangiopericytoma and angiomatous meningioma in both training and validation sets (all P <0.01; **Figure 3**). The fusion radiomic model showed favorable discrimination in predicting the postoperative pathological diagnosis with AUC values of 0.900 (95% CI, 0.879–0.916) and 0.900 (95% CI, 0.879–0.919) in the training and validation sets, respectively (**Figure 1C**). The results of DeLong’s test showed that the fusion radiomic model performed significantly better than the T1 radiomic model (P = 0.013), but there was no significant difference between the fusion radiomic model and T2 radiomic model (P = 0.189).

**Table 4:** Detail information of six selected key radiomic features.

Sequence	Feature name	Feature type	Angiomatous meningioma (n = 73)	Hemangiopericytoma (n = 147)	P-value
T2WI	lbp-3D-k_glrlm ShortRunHighGrayLevelEmphasis	Texture	0.5791 ± 0.0163	0.7400 ± 0.0093	0.0001
	exponential_glcm MaximumProbability	Texture	0.1670 ± 0.0224	0.4673 ± 0.0240	0.0001
	exponential_glrlm_RunVariance	Texture	0.0546 ± 0.0144	0.1784 ± 0.0148	0.0001
	lbp-3D-k firstorder_10Percentile	Firstorder	0.2953 ± 0.0092	0.4000 ± 0.0072	0.0001
	lbp-3D-k firstorder_90Percentile	Firstorder	0.3802 ± 0.0157	0.5754 ± 0.0141	0.0001
CET1	gradient_gldm_Large DependenceHighGrayLevelEmphasis	Texture	0.4053 ± 0.0207	0.2464 ± 0.0088	0.0001

T2WI, T2-weighted imaging; CE-T1WI, contrast-enhanced T1-weighted imaging.

**Figure 4:**
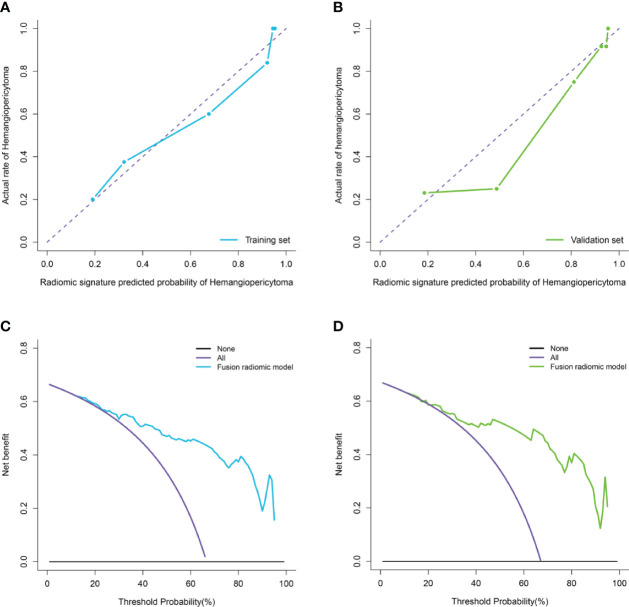
Calibration curve analysis and decision Curve Analysis for the fusion radiomic model. **(A, B)**. Calibration curves of the fusion radiomic model in the training set **(A)** and validation set **(B)**. Calibration curves depict the calibration of model in terms of the agreement between the actual observations and predictions of tumor diagnosis. The Y axis represents the actual rate. The X axis represents the predicted probability. The diagonal purple line represents perfect prediction by an ideal model. The blue **(A)** and green **(B)** lines represent the performance of the model, of which a closer fit to the diagonal purple line represents a better prediction. **(C, D)**. Decision curve analysis for the fusion radiomic model in the training set **(C)** and validation set **(D)**. The Y axis measures the net benefit. The blue **(C)** and green **(D)** line represents the fusion radiomic model. The purple line represents the assumption that all patients were diagnosed as intracranial hemangiopericytoma. The black line represents the assumption that all patients diagnosed as angiomatous meningioma.

Moreover, the calibration curve analysis and Hosmer–Lemeshow test for fusion radiomic model demonstrated good agreement between observations and predictions in the training set (P = 0.211; **Figure 4A**) and the validation set (P = 0.407; **Figure 4B**). The DCA of the fusion radiomic model is presented in **Figures 4C, D**. The fusion radiomic model clearly provided a net benefit over the two schemes, with a threshold probability of >5 and >20% for the training and validation sets, respectively, suggesting the clinical usefulness of the fusion radiomic model.

### Performance of Clinical and Clini-Radiomic Combined Model

The seven available clinical features in the training set were used to build a clinical model based on multivariable logistic regression analysis. We then verified the performance of these models in the validation set. As shown in **Figure 1D**, the AUCs were 0.820 (95% CI, 0.790–0.847) and 0.790 (95% CI, 0.753–0.818) in the training and validation sets, respectively.

**Figure 5:**
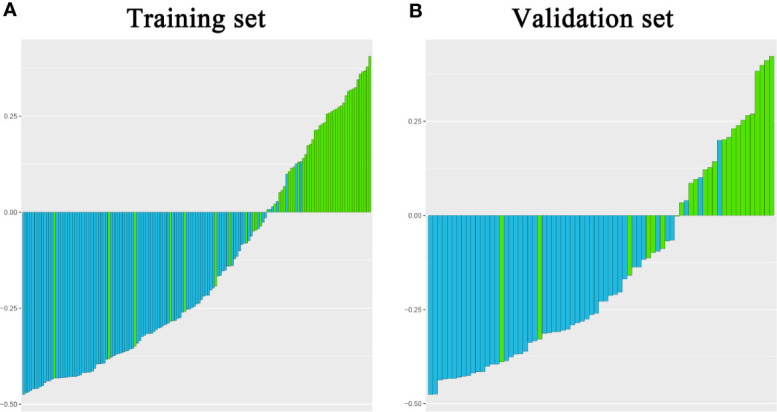
Bar plots for the clini-radiomic combined model in the training **(A)** and validation sets **(B)**. The blue histogram above the horizontal axis and the green histogram below the horizontal axis indicate the patients with correct diagnosis of the clini-radiomic combined model.

**Figure 6:**
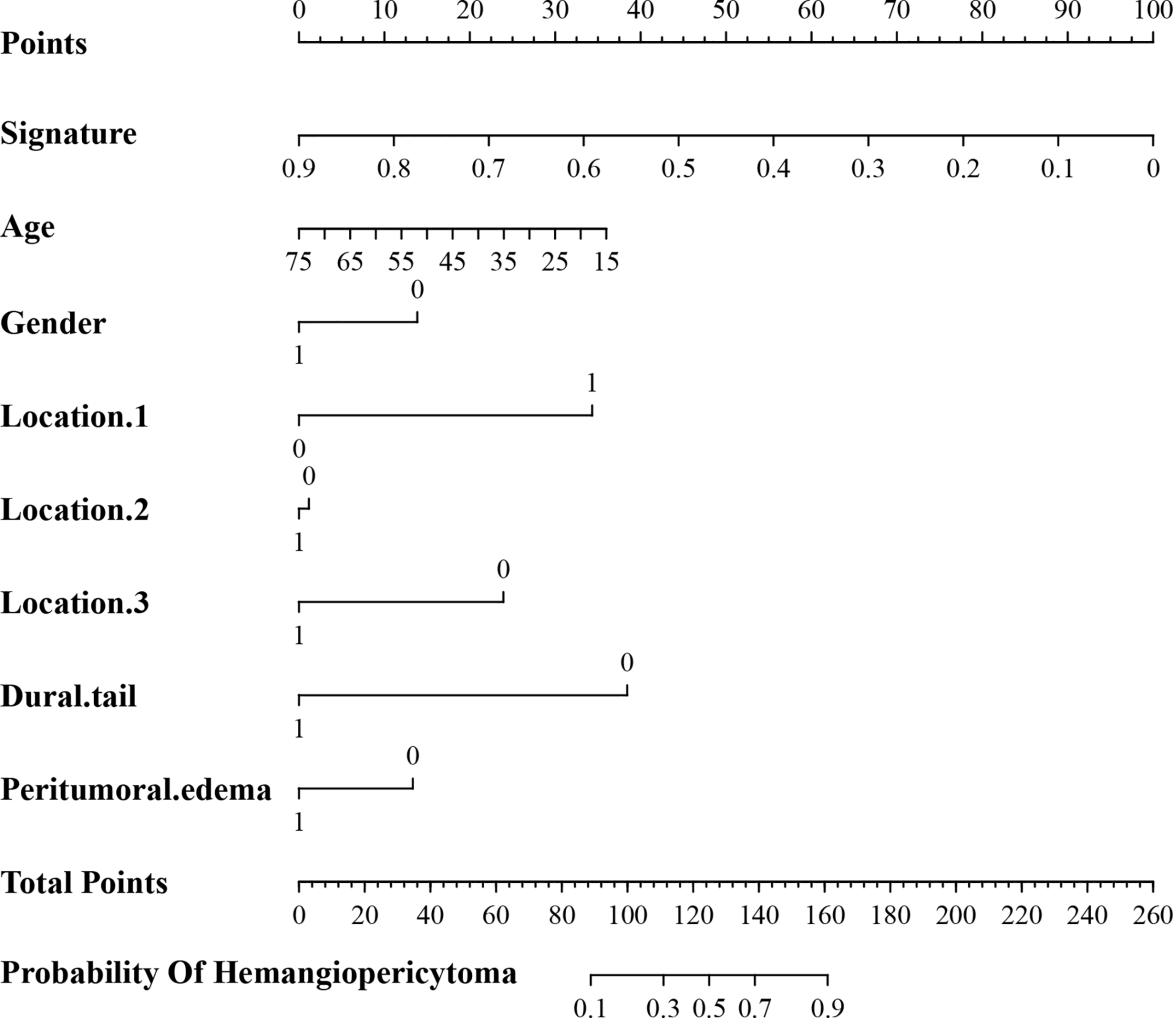
A nomogram derived from the clini-radiomic combined model. This nomogram is used based on the value of signature of radiomic model and clinical characteristics, namely, age, location 1 (supratentorial or infratentorial), location 2 (skull base or non-skull base), location 3 (paravenous sinus or non-paravenous sinus), dural tail, and peritumoral edema. Draw a vertical line from the corresponding axis of each factor until it reaches the first “Points” line. Next, summarize the points of all risk factors, and then draw a vertical line that falls vertically from the “Total Points” axis until it reaches the last axis to the diagnostic probability of intracranial hemangiopericytoma.

In addition, the above clinical model and signature of fusion radiomic model were determined to establish the clini-radiomic combined model, yielded an AUC of 0.920 (95% CI, 0.902–0.942) in the training set and 0.910 (95% CI, 0.894–0.935) in the validation set (**Figure 1E**). The clini-radiomic combined model’s predictive accuracy of tumor diagnosis was 0.884 (0.865–0.905) in the training set and 0.863 (0.842–0.883) in the validation set. The detailed predictive indicators of the aforementioned models are shown in **Table 5**. Bar plots showed the accuracy of clini-radiomic model in the diagnosis of intracranial hemangiopericytoma or angiomatous meningioma (**Figure 5**). As shown in **Figure 6**, the clini-radiomic combined model is presented as a nomogram. The DeLong’s test showed that the clini-radiomic combined model and fusion radiomic model performed significantly better than the clinical model (P <0.01), but there was no significant difference between the clini-radiomic combined model and fusion radiomic model (P = 0.510).

**Table 5:** Details diagnostic ability of all constructed models.

Model	Performance	AUC	ACC	SE	SP	PPV	NPV
T1 radiomic model	Training set	0.840 (0.814–0.863)	0.775 (0.750–0.802)	0.763 (0.731–0.797)	0.796 (0.757–0.837)	0.866 (0.839–0.894)	0.662 (0.617–0.707)
	Validation set	0.750 (0.718–0.788)	0.699 (0.670–0.728)	0.685 (0.651–0.719)	0.737 (0.682–0.792)	0.881 (0.855–0.907)	0.452 (0.403–0.499)
T2 radiomic model	Training set	0.850 (0.829–0.879)	0.701 (0.673–0.729)	0.958 (0.943–0.974)	0.216 (0.173–0.259)	0.697 (0.667–0.727)	0.733 (0.645–0.820)
	Validation set	0.850 (0.828–0.873)	0.726 (0.699–0.754)	0.980 (0.970–0.991)	0.136 (0.098–0.175)	0.725 (0.697–0.753)	0.750 (0.636–0.865)
Clinical model	Training set	0.820 (0.790–0.847)	0.762 (0.735–0.788)	0.857 (0.830–0.884)	0.571 (0.517–0.624)	0.800 (0.770–0.829)	0.667 (0.612–0.722)
	Validation set	0.790 (0.753–0.818)	0.767 (0.741–0.793)	0.796 (0.765–0.826)	0.708 (0.659–0.757)	0.848 (0.820–0.876)	0.630 (0.579–0.678)
Fusion radiomic model	Training set	0.900 (0.879–0.916)	0.810 (0.785–0.834)	0.765 (0.733–0.797)	0.898 (0.866–0.931)	0.938 (0.917–0.959)	0.657 (0.612–0.700)
	Validation set	0.900 (0.879–0.919)	0.822 (0.798–0.846)	0.796 (0.765–0.827)	0.875 (0.840–0.910)	0.929 (0.907–0.950)	0.677 (0.633–0.722)
Clini-radiomic model	Training set	0.920 (0.902–0.942)	0.884 (0.865–0.905)	0.939 (0.921–0.957)	0.776 (0.732–0.821)	0.893 (0.871–0.916)	0.864 (0.825–0.904)
	Validation set	0.910 (0.894–0.935)	0.863 (0.842–0.883)	0.939 (0.921–0.956)	0.708 (0.658–0.757)	0.868 (0.843–0.892)	0.850 (0.809–0.891)

ACC, accuracy; AUC, area under curve; PPV, positive predict value; NPV, negative predictive value.

**Figure 7:**
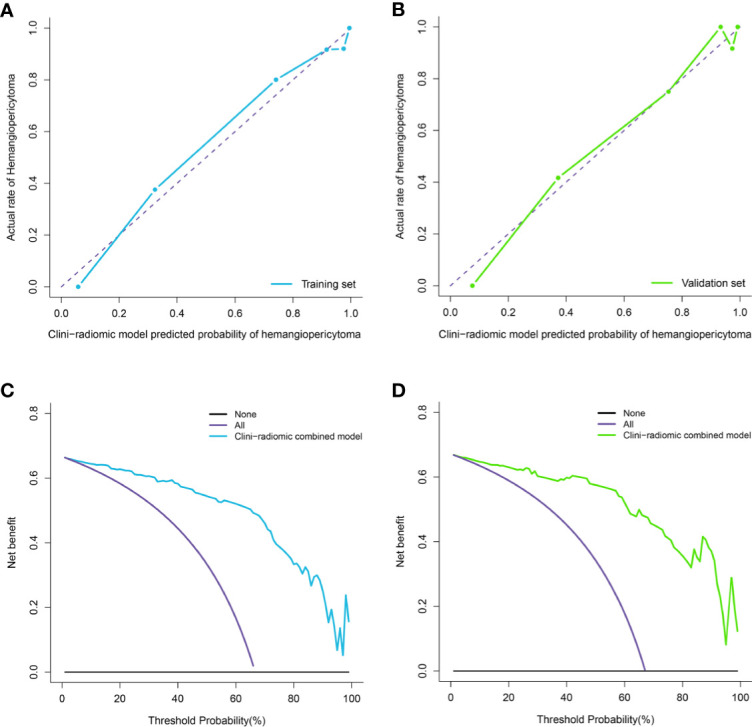
Calibration curve analysis and decision Curve Analysis for the clini-radiomic model. **(A, B)**. Calibration curves of the clini-radiomic model in the training set **(A)** and validation set **(B)**. Calibration curves depict the calibration of model in terms of the agreement between the actual observations and predictions of tumor diagnosis. The Y axis represents the actual rate. The X axis represents the predicted probability. The diagonal purple line represents perfect prediction by an ideal model. The blue **(A)** and green **(B)** lines represent the performance of the model, of which a closer fit to the diagonal purple line represents a better prediction. **(C, D)**. Decision curve analysis for the clini-radiomic model in the training set **(C)** and validation set **(D)**. The Y axis measures the net benefit. The blue **(C)** and green **(D)** line represents the clini-radiomic model. The purple line represents the assumption that all patients were diagnosed as intracranial hemangiopericytoma. The black line represents the assumption that all patients diagnosed as angiomatous meningioma.

The calibration curve analysis, Hosmer–Lemeshow test, and DCA for clini-radiomic combined model are shown in **Figure 7**. The results showed demonstrated good agreement between observations and predictions in both the training (P = 0.240; **Figure 7A**) and validation sets (P = 0.457; **Figure 7B**) for clini-radiomic combined model. The clini-radiomic combined model performed a higher net benefit than both schemes, with a threshold probability of >0% for training set (**Figure 7C**) and >0% for validation set (**Figure 7D**). The results indicating that the clini-radiomic combined model were clinically useful. The decision curve attained better performance for the constructed clini-radiomic model with regard to clinical application.

## Discussion

In this study, we conducted a retrospective analysis of high-quality data from a 10-year cohort of patients with histopathologically confirmed intracranial hemangiopericytoma and angiomatous meningioma, and we used the T2WI and CE-T1WI MRI based radiomic approach to effectively identify intracranial hemangiopericytoma and angiomatous meningioma before operation.

Hemangiopericytoma that originates from the central nervous system is very rare (25). Intracranial hemangiopericytoma was originally classified as hemangioblastic meningioma, and it was later confirmed to be derived from the epithelial cells of meningeal mesenchymal capillaries, rather than meningeal epithelial cells (1). Intracranial hemangiopericytoma is usually isolated and mainly connected to the dura mater, which is attached to the falx or sagittal sinus of the brain, or occurs in the epidural area (26). Intracranial hemangiopericytoma is classified as a malignant tumor (WHO grades II–III) with the tendency of recurrence and metastasis (3). Intracranial hemangiopericytoma exhibits more aggressive behaviors like bone erosions or necrosis, low ADC values and heterogeneous enhancement (27, 28), however, these visual based features are not always effective and reliable. Angiomatous meningioma is a rare WHO grade I meningioma type with a total incidence rate of 2.1–2.59% of all meningiomas (29). The blood supply of angiomatous meningioma is very rich (30), and according to Hasselblatt et al., meningiomas with >50% vascular components can be diagnosed as angiomatous meningioma (14).

In the preoperative radiological examination, compared with other meningiomas, angiomatous meningioma showed more obvious enhancement and vascular signs, and fewer meningeal tail signs, which made it difficult to distinguish angiomatous meningioma from hemangiopericytoma on conventional imaging (30). The imaging characteristics of intracranial hemangiopericytoma and angiomatous meningiomas are similar, but their treatment and prognosis are very different. Intracranial hemangiopericytoma is more aggressive, highly vascularized and prone to intraoperative hemorrhage, has a higher postoperative recurrence rate and a worse prognosis (1). However, angiomatous meningioma is benign and its clinical presentation, surgical management, and prognosis are almost similar to the classical meningioma (2). The surgical resection of angiomatous meningioma is more difficult than other types of meningioma, with more intraoperative bleeding and more serious complications such as neurological impairment (30). Therefore, accurate preoperative diagnosis of intracranial hemangiopericytoma and angiomatous meningioma is of great clinical significance for the planning of operation and the evaluation of prognosis.

Due to the lack of effective molecular markers, the researchers tried to use preoperative images to identify intracranial hemangiopericytoma and angiomatous meningioma. Intracranial hemangiopericytoma have various manifestations in MRI, most of which are irregular or lobulated, with mixed signals and uneven enhancement due to cystic degeneration and necrosis (25). Benign meningiomas have smooth edges, uniform signals, few lobes, and may have signs of calcification and dural tail. However, different from benign meningiomas, MRI features of angiomatous meningiomas are similar to those of intracranial hemangiopericytoma, such as uneven signal, cystic necrosis, irregular lobulation, irregular meningeal tail, etc. Thus, in clinical practice, angiomatous meningiomas and intracranial hemangiopericytoma are difficult to distinguish accurately only by conventional imaging (7). Therefore, an effective, accurate and widely used tool for preoperative identification of angiomatous meningioma and intracranial hemangiopericytoma is in urgent need of development.

As an emerging study field, radiomics can possibly depict the intratumoral heterogeneity based on the quantitative and the classified high-throughput data (31). Typically, novel image-based computational models have played increasingly important roles in the accurate diagnosis and also treatment guidance in neuro-oncology, thanks to the development of clinical imaging data (32). Radiomics has many applications in the central nervous system, such as differential diagnosis (10, 33–35) and classification (12, 15), prediction of molecular characteristics (11, 36), therapeutic response and progress of central nervous system diseases (32, 37). Radiomics mainly uses the following 4 steps to convert image images into mineable data, namely, image acquisition and reconstruction, tumor ROI segmentation, radiomic feature extraction and screening, model construction and verification (38). There is currently a study using radiomics to distinguish intracranial hemangiopericytoma and meningioma before surgery (7), but there are many types of meningiomas, and more attention should be paid to the identification of hemangiopericytoma that is easily confused with intracranial hemangiopericytoma. Meanwhile, previous studies have shown that Li et al. (13) intend to use the radiomic approach of texture analysis to identify intracranial hemangiopericytoma and angiomatous meningioma, but it has many shortcomings. First of all, the number of patients included in the study is small, with only 24 cases intracranial hemangiopericytoma and 43 cases angiomatous meningioma. A smaller number of patients make it more difficult to draw more accurate conclusions. Secondly, the study does not have a corresponding validation set, which limits the clinical applicability of the conclusions. Finally, the study only extracts the texture features of the tumor, and lacks high-latitude radiomic features and more complete model construction methods. Therefore, in the present study, we used the radiomic method to differentiate intracranial hemangiopericytoma from angiomatous meningioma preoperatively.

In the current study, Mann–Whitney U test, elastic net, and RFE algorithm were sequentially utilized to reduce redundant features and select the most appropriate features for the construction of a fusion radiomic model. It is crucial to exclude irrelevant features, because these features may obscure important information and affect the performance of the prediction model (39). First, after the Mann–Whitney U test, we conducted a preliminary screening and obtained 399 radiomic features. Then, 37 radiomic features were further obtained through the elastic net algorithm, and a feasible number that balances insufficient fitting and over fitting is obtained. Finally, 6 features were determined by the RFE algorithm, and the constructed fusion radiomic model achieved balanced performance in both the training [0.900 (95% CI, 0.879–0.916)] and validation [0.900 (95% CI, 0.879–0.919)] sets. Next, the clini-radiomic combined model was constructed in this study, which had incorporated both the fusion radiomic model and the clinical model, with the AUC of 0.920 (95% CI, 0.902–0.942) and 0.910 (95% CI, 0.894–0.935) in training set and validation set, respectively. Both fusion radiomic model and clini-radiomic combined model had displayed good calibration and discrimination. Thirdly, this clini-radiomic combined model was convenient in use, which could accurately differentiate angiomatous meningioma and intracranial hemangiopericytoma before surgery.

Research in intracranial hemangiopericytoma and angiomatous meningioma has been historically limited due to a relatively low incidence, we collected the imaging, clinical and pathological data of 147 cases of intracranial hemangiopericytoma and 73 cases of angiomatous meningioma from a single center for this radiomic research. It is very precious, and the large sample size enrolled in this study will lead to more reliable results than previous scattered case studies. This study also has some limitations. First, this was a single center study, more patients from multiple centers could be used to validate the robustness and repeatability of our clini-radiomic model. Second, prospective studies are necessary to verify the effectiveness and robustness of this clin-radiomics combined model. Thirdly, the research methods of radiomics are various, and different researchers adopt different analyses and preprocessing steps, namely, feature extraction, selection and model construction, so the results may be further optimized.

## Conclusion

Preoperative identification of angiomatous meningioma and intracranial hemangiopericytoma can greatly assist surgery plans making and improve patient prognosis. The clini-radiomic model incorporating the fusion radiomic model and clinical characteristics showed great performance and high sensitivity in the differential diagnoses of angiomatous meningioma and intracranial hemangiopericytoma, and to assist in the development of individualized treatment of patients with angiomatous meningioma and intracranial hemangiopericytoma.

## Data Availability Statement

The raw data supporting the conclusions of this article will be made available by the authors, without undue reservation.

## Ethics Statement

The studies involving human participants were reviewed and approved by the Beijing Tiantan Hospital. Written informed consent for participation was not required for this study in accordance with the national legislation and the institutional requirements.

## Author Contributions

YF and PL revised the manuscript for important intellectual content. JZ, ZW, and LW take final responsibility for this article. All authors analyzed and interpreted the data. All authors contributed to the article and approved the submitted version.

## Funding

This work was supported by the Natural Science Foundation of China (Grant No: 82102144, 81472370 and 81672506) and the Natural Science Foundation of Beijing (Grant No. 7192056 and J180005).

## Conflict of Interest

The authors declare that the research was conducted in the absence of any commercial or financial relationships that could be construed as a potential conflict of interest.

## Publisher’s Note

All claims expressed in this article are solely those of the authors and do not necessarily represent those of their affiliated organizations, or those of the publisher, the editors and the reviewers. Any product that may be evaluated in this article, or claim that may be made by its manufacturer, is not guaranteed or endorsed by the publisher.

## Abbreviations

HPC, hemangiopericytoma; T2WI, T2-weighted imaging; CE-T1WI, contrast-enhanced T1-weighted imaging; ROI, regions of interest; GLCM, gray level co-occurrence matrix; GLRLM, gray level run-length matrix; LASSO, least absolute shrinkage and selection operator; RFE, recursive feature elimination; ROC, receiver operating characteristic; AIC, Akaike information criterion; AUC, area under curve; DCA, Decision curve analysis; ACC, accuracy; PPV, positive predictive value; NPV, negative predictive value.
